# Using photographs for rating severity degrees of clinical appearance in research mice enables valid discrimination of extreme but not mild and moderate conditions: A pilot study

**DOI:** 10.1371/journal.pone.0287965

**Published:** 2023-11-02

**Authors:** Johanne C. Krueger, Maren Boecker, Siegfried Gauggel, Andre Bleich, Rene H. Tolba

**Affiliations:** 1 Institute for Laboratory Animal Science and Experimental Surgery, RWTH Aachen University, Faculty of Medicine, Aachen, Germany; 2 Animal Welfare Unit, University of Bonn, Bonn, Germany; 3 Institute for Medical Psychology and Medical Sociology, RWTH Aachen University, Faculty of Medicine, Aachen, Germany; 4 Institute for Laboratory Animal Science, Hannover Medical School, Hannover, Germany; University of Birmingham, UNITED KINGDOM

## Abstract

To ensure good animal welfare in laboratory research and in stockbreeding severity ratings of the animals´ wellbeing are essential. The current study investigated how valid raters can evaluate different severity degrees of clinical appearance and how ratings might be influenced by factors other than the severity itself. Ninety-seven people rated the severity degree (none, mild, moderate, or severe) of the clinical appearance of mice seen in eight different images. The images also differed in the perspective in which they had been taken (entire mouse or head only). The raters differed with regard to their experience of working with laboratory animals and were subsequently divided into three groups—beginners, advanced, professionals. Generalisability theory was applied to examine the contribution of the different rater (raters themselves and experience) and image facets (actual degree of severity and perspective) to the overall data variability. The images showing the extreme severity degrees were rated more homogenously and more precisely than were the images showing the intermediate degrees, as compared to the reference scores. The largest source of variance was the actual degree of severity, accounting for 56.6% of the total variance. Considering only the images showing the extreme severity degrees, this percentage rose to 91.6%, accounting almost exclusively for the found variance. In considering only the intermediate severity degrees, the actual degree of severity did not contribute to variance at all. The remaining variance was due to the raters and the interactions between raters, the actual degree of severity and the perspective. The experience of the raters did not account for any variance. Training in the assessment of severity degrees seems necessary to enhance detection of the intermediate degrees of severity, especially when images are used. In addition, good training material should be developed and evaluated to optimise teaching and to minimise wrong assessments.

## Introduction

The use of laboratory animals is an inherent part of research in medicine. Nevertheless, there have been heated debates for many years regarding this topic. Discussions are influenced by moral and emotional aspects, of which the core question is whether humans are justified to harm an animal, causing pain, suffering or distress in order to benefit their own species.

The protection of animals and ensuring good animal welfare, defined as the ‘physical and mental aspects of quality of life and extending beyond the absence of disease’ [1, 2], has become one of the constitutional aims in Germany and is also indicated in the European Directive 2010/63. As defined in the European Directive, institutions are obliged to carry out severity classifications of procedures ´on the basis of estimated levels of pain, suffering, distress and lasting harm´ being inflicted on the animals [3]. Moreover, not only these estimated (prospective) levels shall be assigned to the different procedures, but the entity of anxiety, affective internal/emotional state as well as the ´actual severity of the pain, suffering, distress and lasting harm´ experienced by the animal [3, 4] shown through varying reactions of the experimental insult or stimuli. The classification of the procedures´ severity being inflicted on the animal as well as their reaction towards that stimuli are divided into: ´non-recovery´, ´mild´, ´moderate´, and ´severe´. Morton [5] recently suggested not only to classify a procedure´s severity before conducting the experiment, but to standardly evaluate throughout and after the experiment whether the severity classification had been appropriately chosen with regards to the severity actually seen in the animals during the conduction of the experiment.

To determine and detect changes in the animals´ wellbeing there are different methods available (e.g. through deviations in physiology, clinic; behavioural changes; alteration of biochemistry, biomarkers) [4]. Morton and Griffiths [6] first described a scheme, with species specific parameters and certain assigned scores. Some of the parameters were objective, meaning that they can be directly measured leaving no scope for interpretation (e.g. heart rate, body weight), whereas others were more subjective such as appearance and behaviour [6, 7]. These latter have to be rated and their rating might not only depend on the actual appearance and behaviour but also on characteristics of the raters themselves (e.g. perception and interpretation because of the experience with the species, overall experience with animal experiments or personal bias) [8–10] or on the material and setting used for assessment (assessment instrument, live observation, video or photo material). Regarding live observation, the presence of the raters might change the behaviour of the animals—causing distress on the animals, as well as prey animals might disguise their behaviour in the presence of humans [1, 11, 12].

Another problem associated with ratings of the animals´ wellbeing is that not all severity degrees might be equally easy to assess correctly and consistently, especially when focusing on subjective parameters. In the literature, there are a few studies available on the assessment of different severity degrees. They consistently show that the extremes of a scale are more likely to be detected correctly than are the degrees in between [6, 13]. Moreover, e.g. when dealing with locomotion scores in cows, slight deviations in the grading are not well recognized [14–16]. Schlageter-Tello et al. [14] showed that in a Likert-type scale with five rating categories, the intermediate levels had low intra- and inter-observer agreement, in contrast to the extreme levels of the grading ranging from no to severe. Thus, cows showing slight deviations in the gait were difficult to detect. Also, Garcia et al. [13] investigated mobility scoring in dairy cows, showing that the highest agreement in the ratings was achieved in the extremes of the scale.

Therefore, we hypothesised that when assessing clinical appearance in laboratory animals, namely mice, the extremes on the scale, ‘none’ and ‘severe’, could be detected more consistently and precisely than could the severity degrees in between (‘mild’ and ‘moderate’). However, to disentangle the contribution of different sources of variance from the severity ratings of clinical appearance, characteristics of the images and raters were systematically combined: people with varying degrees of experience with laboratory animals were asked to rate the degree of severity of photographed mice according to the varying categories ‘mild’, ‘moderate’, ‘severe’ and additionally ‘none’ for an animal without any deviation from its normal state.

## Material and methods

The study was ethically approved by the local ethics committee (*Ethik-Kommission der Medizinischen Fakultät der Rheinisch-Westfälischen Technischen Hochschule Aachen)* under the internal number EK 230/22.

### Rater

Ninety-seven people from six different laboratory animal science related locations throughout Germany were asked to participate in the study and rated the severity degree of the clinical appearance of eight photographed mice. Each respondent completed and returned a printed version of the questionnaire (S1 Questionnaire). No payment or other incentive was given.

The study was performed during January and February 2019. In total 97 raters, of which 45.4% (44/97) were male and 54.6% (53/97) female, participated.

For practical reasons regarding the application of the G-theory software for which equal-sized rater experience groups were needed, 13 raters had to be excluded from further analyses, leaving a final total of 84 raters evaluated in the study (more details can be found in the Statistical Methods section).

Besides the severity rating of the mice, the raters had to respond to a few sociodemographic questions, including questions related to laboratory animal experience, since the raters, had different backgrounds in working with laboratory animals regarding profession and work experience.

These questions focused on differentiating between whether participation in animal experiments was carried out, the involvement in taking care of animals and the experience with types of animals (large, rodents or both) (S1 Questionnaire). Based on the response to these questions, the participants were divided by the study supervisor into three different categories, namely, ‘beginners’ (B; n = 37, but reduced to n = 28 [male: n = 13 (46.4%); female: n = 15 (53.6%)]), ´advanced’ (A; n = 32, but reduced to n = 28 [male: n = 13 (46.4%); female: n = 15 (53.6%)]), and ‘professionals’ (P; n = 28 [male: n = 10 (35.7%); female: n = 18 (64.3%)]) (Table 1).

**Table 1:** Classification of raters by using their self-declared answers regarding participation in animal experiments/care taking as well as on animal-handling experience with different species (large laboratory animals and rats and mice) into three consecutive groups (B: Beginners, A: Advanced, P: Professionals).

Participation in Animal Experiments	Participation in Animal Care Taking	Experience with Large Laboratory Animals	Experience with Rats and Mice	Rater Category	n	Cumulative n
<1 year	<1 year	Yes or no	Yes or no	B	28	28
1–5 years	<5 years	Yes or no	Yes	A	10	28
<5 years	1–5 years	Yes or no	Yes	A	16	
<5 years	1–5 years	Yes	No	A	1	
>5 years	<5 years	Yes	No	A	1	
>5 years	Irrelevant*	Yes or no	Yes	P	11	28
Irrelevant**	>5 years	Yes or no	Yes	P	17	

B, beginners; A, advanced; P, professionals;

*Because of their long experience in animal experiments, people were classified as professionals regardless of their amount of experience in animal care taking.

** Because of their long experience in animal care, people were classified as professionals regardless of their amount of experience in animal experiments.

### Images

**Fig 1:**
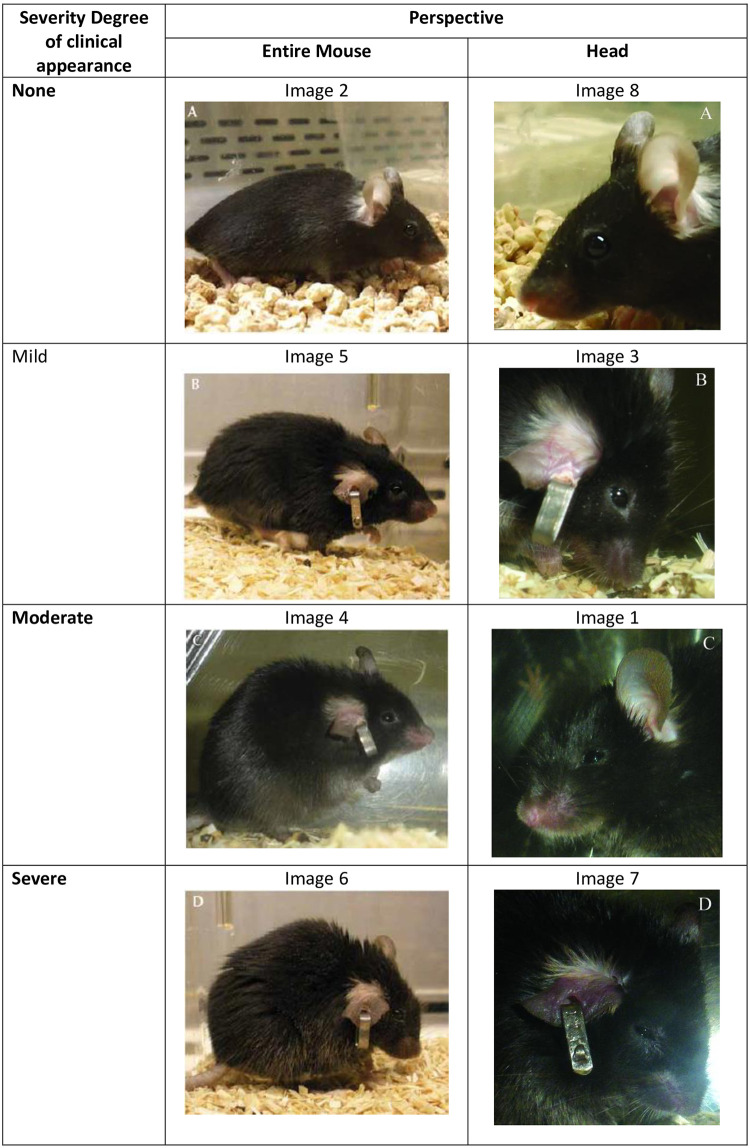
Photographs of mice used to assess different severity degrees of clinical appearance in a questionnaire. The position of the images was assigned randomly. The numbers shown in the Table match the position of the photograph in the questionnaire.

Eight different images of mice (Fig 1) were presented for evaluation to the raters. The images were taken from the publication, ‘Predictive Observation-Based Endpoint Criteria for Mice Receiving Total Body Irradiation’ [17], with the permission of the publishers of the *Journal of Comparative Medicine*. The eight images varied according to two attributes: the severity of the clinical appearance of the pictured mouse and the perspective in which the respective mouse was photographed. Regarding the actual degree of severity shown in the pictured mouse, four different degrees were visualised on the images (none, mild, moderate and severe). Each degree of severity was displayed twice–once with images of the entire mouse and another time with images showing just the head of the mouse.

The actual degree shown in the pictures of the mice was determined as follows: Two laboratory animal experts, one of them with over 20 years of experience, the other being lab animal veterinarian, evaluated each image in consensus. Therefore, the original parameters assigned [17] were used to get a first impression (proof of concept) and one additional parameter (orbital tightening) was added to get an overall score. However, the literature has provided numerous ways to assess and grade the clinical presentation of mice [6, 17–19]. Therefore, the two experts chose two additional parameters (Mouse Grimace Scale [MGS] and appearance; supplementary material [S1 Table]) to determine the actual degree of severity shown in the images. All parameters were combined, and the overall degree of severity (1–none, 2–mild, 3–moderate, and 4–severe) was assigned to each mouse seen in the images. The agreed-on results, ‘reference scores’ from here onwards, are shown in Fig 1. The parameters used to assign the degree of severity are presented in S1 Table.

The letters seen on the images (Fig 1) were covered by a white square to avoid prediction and expectation bias regarding the degree of severity. The raters were asked to rate the degree of severity for each of the images using a 4-point rating scale (1 = none, 2 = mild, 3 = moderate and 4 = severe). They were not informed about how many times each degree of severity was displayed, neither of the parameters used to score the animals. This was part of the study design in order to clarify their intuitive evaluation. The instructions of the questionnaire read as: “Please select one of the following terms, describing the severity of the animals shown in the pictures below. Please only choose one term for each picture: “Non”; “Mild”; “Moderate”; “Severe”” (S1 Questionnaire). The order of the images in the questionnaire was randomised using the randomizing software available under http://www.randomizer.org/ [20].

## Statistical methods

Descriptive statistical analysis was performed. Means (M) and standard deviations (SDs) were derived for each image and rater group separately to get an overall impression of the data distribution and potential deviations from the respective reference score. P-values were computed using one-way ANOVA. Additionally, a boxplot diagram showing the score distributions for each image across all raters was created.

As it had been assumed that it might be easier to rate the extremes of the scale as compared to the middle degrees of severity, the mean absolute deviations from the reference scores and their SDs were calculated. Levene´s test was performed to test for variance homogeneity. Welch-test was performed to compare the mean absolute deviations of the images showing the extremes of the scale as compared with the intermediate ones. SPSS Statistics Version 25 (IBM Corporation, Armonk, NY, USA) was used for statistics and graphing.

### Generalisability theory

In the present study, generalisability analyses were conducted to estimate the contribution of different sources of variance to the severity ratings of the pictured mice (Table 2) [21, 22]. Generalisability theory (G-theory) can be seen as an extension of classical test theory, enabling the researcher to deal with multiple sources of errors simultaneously. The EduG 6.1 software [23] was employed for this purpose. Before the analyses were conducted, 13 raters had to be excluded. This was done as the facet ‘rater’ was nested in the facet ‘experience group’, and analysis with the EduG 6.1 software required that each group had the same number of raters. Consequently, the number of raters of the B- and A-groups had to be reduced to 28, which was the number of the smallest group, the P-group. As one of the research questions of the present study was whether the experience of the raters influenced the severity rating of the pictured mice, the raters to be excluded were selected in such a way that the difference between the experience groups was maximised (e.g. the nine raters of the A-group with the most experience were excluded). Additionally, a second line of analyses was conducted, this time with the 28 raters of the A- and T-groups randomly drawn. The results of the latter analyses will not be reported, as no differences were obtained.

**Table 2:** Potential factors influencing the rating of the clinical presentation in photographs of mice investigated with generalisability study.

	Facet Effect (With Facet Levels)	Meaning (If Facet Accounts Substantially for Variance)
**Characteristics of images**	Severity (S) (none, mild, moderate, severe)	The actual degree of severity shown by the pictured mouse^2^ influences the severity rating of the pictured mouse. (This should be by far the largest source of variance.)
	Perspective (P) (full body, head)	The perspective in which the mouse is presented influences the severity rating of the pictured mouse.
**Characteristics of raters**	Rater experience (E) (beginner, advanced, professional)	The degree of experience regarding the work with mice influences the severity rating of the pictured mouse.
	Rater (R)^1^	The severity rating of the pictured mouse varies across the raters. (A very small variance component for the R:E facet would reflect a high inter-rater reliability.)

^**1**^Nested in experience group, as each rater can only belong to one experience group;

^2^as rated by experts (see the Material and Methods section).

As described above, the raters had to judge the degree of severity in each of the eight images using a Likert-type 4-point rating scale (1 = none, 2 = mild, 3 = moderate and 4 = severe). The images differed according to the two facets, the ‘actual severity degree of clinical appearance of the pictured mouse’ (S; 4 levels) and ‘perspective’ (P; 2 levels) (Table 2), such that each S × P combination was presented by one image. The raters differed in terms of ‘experience’ (E; 3 levels) and the ‘raters’ themselves, which were nested in the experience groups (R:E; 28 levels). A random-effect nested measurement design (S × P × E × R:E) was applied where S, P and E were treated as fixed and R:E as random.

Variance components were computed for each of the facets and for their interactions. EduG estimated the variance components by applying a Whimbey’s correction to conventional ANOVA estimates, which accounts for the type of sampling involved (i.e. random, fixed or random finite). This calculation of variance components determined how much variance is due to the actual degree of severity of the pictured mice, the perspective the photo was taken, the raters’ experience level and the raters themselves. This procedure was first carried out for all eight images and subsequently in two further and separate analyses for the extreme severity levels and the middle severity levels.

## Results

### Descriptive statistics for the images

**Fig 2:**
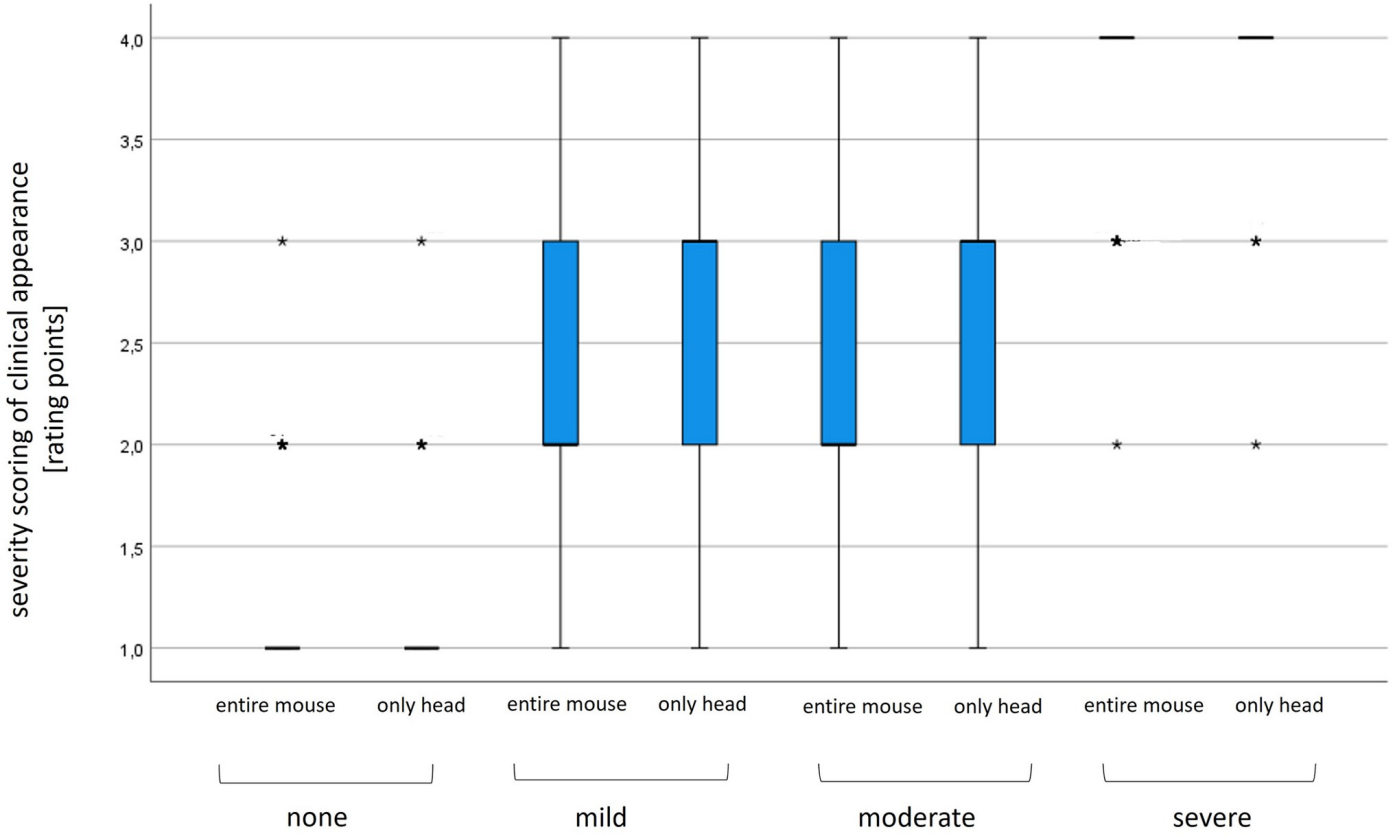
Box and whisker plot for all severity ratings of clinic appearance. Results are presented over all raters (n = 84) regardless of their laboratory animal experience and for each picture individually. *Single ratings of raters.

The descriptive statistics for each image and experience group are presented in Table 3. No substantial differences were observed among the different rater groups in terms of the mean severity ratings. A significant difference between the groups for each image was only found for Image 7 (p = 0.02). The mean absolute deviations from the reference scores across all raters varied between 0.06 (Image 7) and 0.25 (Image 6) for the images showing the extremes none and severe), whereas it differed between 0.56 (Image 5) and 0.89 (Image 3) for the images showing intermediate (mild and moderate) degrees of severity. The Levene´s test revealed variance heterogeneity for the mean deviations from the reference scores regarding the ratings of the extreme compared to the intermediate degrees of severity (p < 0.000). Thus, a Welch test was performed for further analysis, showing a significant difference (p < 0.000). Fig 2 also shows that the middle categories were rated less correctly and also less consistently, as indicated by the higher variance found for the intermediate-degree images.

**Table 3:** Results of clinical appearance severity scoring based on rater experience presented as mean, Standard Deviation (SD), p-values and absolute deviation from the reference score.

Degree of Severity	Perspective	Beginners (n = 28)		Advanced (n = 28)		Professionals (n = 28)		p-value (ANOVA)	Absolute Deviation from the Reference Score	
		Mean	SD	Mean	SD	Mean	SD		Mean	SD
None	Entire mouse	1.25	0.44	1.21	0.50	1.14	0.36	0.65	0.20	0.43
	Head	1.18	0.48	1.07	0.26	1.00	0.00	0.11	0.08	0.32
Mild	Entire mouse	2.21	0.63	2.21	0.83	2.46	0.74	0.35	0.56	0.57
	Head	2.89	0.96	2.71	0.71	2.86	0.80	0.70	0.89	0.74
Moderate	Entire mouse	2.29	0.76	2.32	0.77	2.18	0.61	0.74	0.81	0.63
	Head	2.93	0.77	2.89	0.83	2.71	0.94	0.60	0.58	0.62
Severe	Entire mouse	3.71	0.54	3.79	0.42	3.75	0.44	0.85	0.25	0.46
	Head	3.82	0.48	4.00	0.00	4.00	0.00	0.02	0.06	0.28

### Generalisability theory

The results of the descriptive statistics were strongly supported by the generalisability analyses (Table 4). Differences from the overall grand mean, which is the mean rating across all images and raters, were highest in the severity (S) facet, followed by the rater nested in the experience groups (R:E) facet. The differences were negligible for the experience (E) facet. The significant differences in the severity facet were especially due to the relatively more accurate scoring and differentiation of the extreme severity levels from the ‘mild’ and ‘moderate’ levels. The raters were not able to differentiate distinctly between the latter two levels.

**Table 4:** Descriptive statistics of the main effects (severity degree of clinical appearance of pictured mouse, perspective, experience, and rater) when rating the degree of severity of clinical appearance in pictured mice analysed using generalisability theory.

**Main effects**			**Mean (SD)**	**Δ**_**to grand mean**_
		Grand mean	2.53 (1.15)	0
	**Severity degree of clinical appearance of pictured mouse**	None (1)	1.14 (.38)	**1.39**
		Mild (2)	2.56 (.82)	−0.03
		Moderate (3)	2.55 (.83)	−0.02
		Severe	3.85 (.39)	**−1.32**
	**Perspective**	Entire mouse	2.38 (1.09)	0.15
		Head	2.67 (1.20)	−0.14
	**Experience**	Beginner	2.54 (1.13)	−0.01
		Advanced	2.53 (1.16)	0
		Professional	2.51 (1.16)	0.02
	**Rater** *	Rater_min_	1.88 (1.05)	**0.65**
		…………		
		Rater_max_	3.13 (1.27)	**−0.60**

SD, standard deviation; Δ, difference; bold, values of special interest.

*As there were 84 raters only the two raters with either the minimum or maximum mean scores across the eight images are shown with both having the biggest Δ to grand mean.

The results of the generalisability analyses are summarised in S2A Table for the rating of all eight images. It includes the classical ANOVA estimates for the severity, perspective, experience and rater experience group facets and their interactions. The ‘%’ column shows the proportion of the variance that is attributable to each of the different sources of variance. As expected and desired, by far the largest degree of data variance was due to the severity (S facet;56.3%). Although the extent of experience (E facet) did not contribute at all to the variance, 5.3% could be attributed to the rater experience group (R:E facet). An additional 11.6%, 5.9% and 18.1% were attributed to each of the interactions of the rater (R facet) with the severity facet and perspective facet and to the multiple interactions of the rater experience group, severity and perspective (R:E, S and P) facets, respectively.

The results of the separate analyses for the extreme severity levels (none and severe) and the middle severity levels (mild and moderate) are also shown in S2B and S2C Table. Although in the analysis of the extreme severity levels the severity facet was predominantly the unique source of variance (91.6%), it did not contribute at all to the variance in the middle severity levels. Here the variance was due to the rater experience group facet (R:E 13.2%), perspective facet (6.6%) and the interactions of the rater experience group (R:E) facet with the perspective facet (15.6%) and severity facet (23%). As in the analysis for all eight images, the extent of experience (E facet) did not contribute at all to the variance in both analyses.

## Discussion

The aim of the study was to determine how well the different degrees of severity can be differentiated by raters and whether the extremes of a rating scale are easier to determine than are the intermediate degrees. Additionally, we investigated the extent that the rating might be influenced by factors other than the presented degree of severity. These factors included the characteristics of the raters (experience) and of the items being rated (perspective of the mice on the images), as well as their interactions.

The extremes of the scale were found easier to rate, and if only the extreme levels were considered, the actual degree of severity was almost entirely the unique source of variance. However, the degree of severity did not contribute to the variance in the intermediate degrees at all. Furthermore, the raters’ experience had no influence on the rating, only in combination with other facets.

The study confirmed a higher homogeneity for the ratings of the extreme degrees of the scale (none and severe) compared with the intermediate degrees of severity (mild and moderate) and a smaller deviation from the reference scores. These findings were also supported by the generalisability analysis, where the resulting variance of the ratings of the extremes of the severity scale was nearly completely due to the degree of severity itself (Severity facet; 91.6%). In contrast, the severity degree of clinical appearance of the pictured mouse did not contribute to the variance in the ratings of the intermediate degrees of severity. Here, the interaction between the different facets was decisive, as well as the facet of the raters nested in experience and the facet of the perspective. These results are in line with previous research, especially with regard to farm animals, since no literature on laboratory animals was found. Garcia et al. [13] found that in a video-based mobility scoring of dairy cows, the extremes of the scoring scale were easier to assess than were the grades in between. In addition, clinically detectable lame animals were more easily identified by most of the raters. In contrast, a differentiation between physiological gait and subclinical altered moving patterns seems to be more difficult [16]. This indicates that slight alterations to the normal state usually being scored in the grades in between the extremes are more difficult to be scored precisely and consistently even by experienced or trained raters [14, 15].

Focusing on the influencing factors upon the ratings, the generalisability analysis indicated that the actual degree of severity shown in the pictured mice was as expected and, as it should be, by far, the biggest source of variance when all eight images were analysed. Other sources of variance were the raters themselves, interactions between the raters, the actual degree of severity of the pictured mice and the perspective in which the photograph was taken. The variance due to the raters and the interactions of severity and perspective with raters is attributed to the severity ratings of the photos showing the intermediate degrees of severity.

Rather unexpectedly, the perspective and its interactions also contributed to variance. Overall, the images showing only the head of the mouse were rated slightly higher regarding the severity of clinical appearance than were the photos of the same severity degree of clinical appearance showing the entire mouse. This effect was particularly pronounced for the analysis of the intermediate levels of severity. One influencing factor might have been the earclip being much more dominant in the photographs only showing the head. Raters were not familiar with this labelling method and might have defined this treatment as inappropriate and welfare relevant. This might also have influenced the ratings, since the ear position was altered because of the metal clip. The effect of focusing on one specific criterion only, in this case the ear clip, and drawing conclusions about the overall well-being of the animal seems to be a very human way of seeing things. In psychology, it is known as the halo effect, meaning that a criterion induces an assumption of how the rest (of the animal) has to look like and might lead, to a false impression of how the subject is burdened [24, 25]. This might also have been the case in this study because of the negation of the parameters ‘fur appearance’ and ‘body position’, which were not shown in detail, and the specific focus on the head of the animal.

Interestingly, the experience of the raters did not contribute to the variance in any of the analyses. Renner et al. [26] found that differences in behaviour of rats living in either enriched or in impoverished environments, were detected equally successful, regardless of the raters´ experience. In line with these findings, Garcia et al. [13] showed that, in assessing cow lameness, within-observer agreements of rating did not differ, even after training, between experienced and inexperienced raters.

Nevertheless, experience and training might be useful in perceiving subtler variations in physical conditions [27], as well as for pain scoring [28] and data gathering [26]. Additionally, since the ratings are linked to what the observer expects, the observers’ range of experience seems to be important [8]. Controversially, this concept was not supported by the present study, likely because of the sole use of photographs, as well as the lacking information regarding the parameters accounting for the severity assessment.

This draws the attention to the limitations of this study. First is the sole use of images instead of including living mice or videos. When thinking about material to use for these kind of studies and as training material, different circumstances must be considered. The most important one seems to be the unethical nature of conducting animal experiments related to harmful procedures where the animals show specific and repeatable signs of deviations from normal appearance just for training purposes. Also, this would not support the ethical aspect of protecting laboratory animals and would be contrary to the ´3R principles´ [29]. However, when using images or videos, one has to focus on the quality of the material used. In our case, we found some images that were not as good as the other ones because of improper illumination or glare (‘moderate’ images of the whole mouse and ‘severe’ images of the head of the mouse). These factors might influence the ratings, besides the perspective itself. The use of video sequences was also discussed in other publications assessing animal welfare statuses of sows and sheep [30, 31]. Nevertheless, many authors use photographs in assessing severity, especially in mice [17, 18]. Foddai et. al. [32] reported that scores assigned to photographs were even less variable than were those from video sequences. Miller et al. found that live scores are significantly lower than scores from images [33]. When comparing videos with photographs some clinical signs would be more appropriately and accurately rated with videos, e.g. gait, some dynamic behaviours such as epilepsy, speed of ambulation and other movements. However, as demonstrated here other clinical signs can also be scored quite accurately with photographs [33].

Another drawback regarding the used material was the use of photographs depicting black-coloured mice since whisker position might be more difficult to score [34]. However, this effect might be neglectable when using good contrasting backgrounds in photographs or recordings [18]. It has been suggested that a more accurate score may be achieved in the Mouse Grimace Scale in animals with dark coat colours [35].

Another limitation of this study is the assignment to the experience groups. The question arises as to how a beginner is defined. Even people who have worked less than a year with animals might have a better knowledge than people working in the field for up to 5 years which may be related to the actual time spent with animals rather than to the time spent in a job (e.g., checking on the animals and caring for them on a regular basis such as animal caretakers or post-graduate students). We recommend assessing the frequency of animal contact in future studies. Combined with years of experience, this would give a better impression of the experience level and might have a different impact on the ratings.

In addition, another influence on the rating might be background information on the parameters rated. In the present study, raters were deliberately left without information regarding the parameters used for the reference scores (S1 Table), and only the overall degree of severity was given. The purpose was to get an unbiased impression on how well the degrees of severity can be determined without any prior instructions and to widen the effect of experience that might have been found throughout the different groups.

Knowing the parameters on which the assessment should be based beforehand may produce a more precise outcome in rating the intermediate degrees of severity. The definition of terms and training previously showed an improvement in standardisation [27] and scoring [36]. A method which could also be used for defining appropriate parameters is the Qualitative Behaviour Assessment, which was e.g. used by Wemelsfelder et al. in cattle [37]. Formal training programmes are especially recommended for animal welfare assessors to reduce inter- and intra-observer variation when focusing on animal-based measures [38] and to increase reliability and agreement among raters [10, 39]. This goes in line with the results of the present study, since we strongly suggest that people working with animals have to be trained to detect and categorize mild to moderately affected mice based on their clinical appearance reliably. A huge impact on animal welfare could be obtained, since early intervention can prevent animals from reaching a ‘severe’ state. That way we could reduce the animals’ pain, distress or lasting harm and would contribute to refine animal experiments as stated in ´3R principles´ [29]. Also, the comparison of the severity experienced by an animal or an interventional cohort and the severity classification of that model given in the EU Directive would benefit to the animals´ welfare, since humane endpoints have to be applied once the severity is exceeded. Therefore, a new semiquantitative assessment has been developed by Morton [5]. Overall, the accurate ratings of degrees of severity need closer attention from the community and clearer parameters on which the rating decisions should be based. Furthermore, parameters, which are to be evaluated when rating the degrees of severity of animals, have to be known and clearly defined [5]. Regular teaching and training sessions are necessary and required to maintain good animal welfare, regardless of the background experience.

## Conclusion

This pilot study used photographed mice showing different degrees of severity in their clinical appearance to evaluate the rating behaviour of people with different expertise in laboratory animal science (beginners, advanced, professionals). The grading of (un)altered clinical appearance (none, mild, moderate, severe) resulted in highly accurate ratings for the extremes (none and severe) but considerable variability for intermediate degrees (mild and moderate). Therefore, we suggest further education, training and overall deepening of people’s knowledge about animal behaviour and appearance. Training outcomes need to be tested and retested so that a certain standard is maintained and raters do not incorporate their own expectations or individual interpretations of the criteria [40]. Further research is necessary to determine whether improvements in detailed grade explanation of scores and additional objectivity-based items should be added to differentiate the degree of severity in animal experiments more precisely, especially among intermediate degrees of severity.

## Supporting information

S1 Questionnaire (DOCX)

S1 Table Variables comprising the reference scores. (DOCX)

S2 Table ANOVA for the rating of the different degrees of severity of clinical appearance in images of mice, using the S x P x E x R:E design of the G theory with interactions. (DOCX)
