# Supplementary material for: Using photographs for rating severity degrees of clinical appearance in research mice enables valid discrimination of extreme but not mild and moderate conditions: A pilot study
Source: PLoS One. 2023 Nov 2;18(11):e0287965. doi: 10.1371/journal.pone.0287965 (PMC10621849; doi:10.1371/journal.pone.0287965)
Supplement: S1 Questionnaire — (DOCX) [file pone.0287965.s001.docx]

**Supplementary data:**

**S1: Questionnaire**

**Questionnaire – observation-based severity in mice**

**Code of your questionnaire:**

Please answer the following questions and put the answers (letters or numbers) on the assign lines

1. First letter of your first name
2. Month in which you were born (“01” for January, “02” for February and so on, “12” for December)
3. First letter of your surname
4. First letter of your place of birth

___ ___ ___ ___

1. 2. 3. 4.

**Part A:** Please select the answers that apply to you:

1. Please select your gender:

- female
- male

1. What is your profession?

____________________________________________________________

1. How long have you been working in laboratory animal science?

- no experience
- < 1 year
- 1 – 5 years
- > 5 years

1. For how many years have you been practically participating in animal experiments?

- No experience
- < 1 year
- 1 – 5 years
- > 5 years

1. I am participating in the care of the animal (similar to an animal technician)

- No
- < 1 year
- 1 – 5 years
- > 5 years

1. If your practical experience with animal experiments or the care of animals is in the past – How many years is this ago?

__________________________________________________________

1. I am working/worked with large animals (i.e.: pig, sheep, calves, etc.).:

- Yes
- No

1. I am working/worked with mice and/or rats.:

- Yes
- No

**Part B:** Please select one of the following terms, describing the severity of the animals shown in the pictures below. Please only choose one term for each picture:

- “Non”
- “Mild”
- “Moderate”
- “Severe”


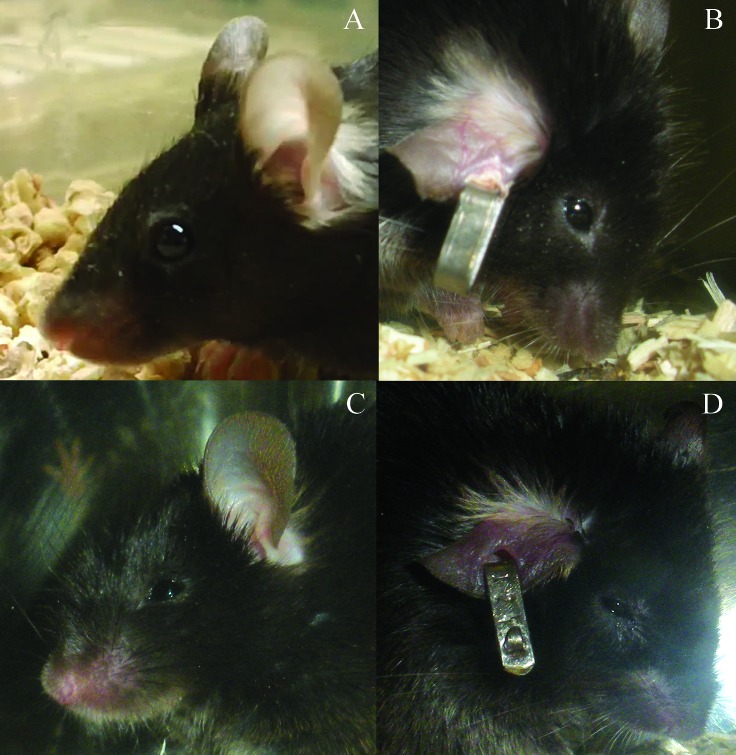


- Non
- Mild
- Moderate
- Severe


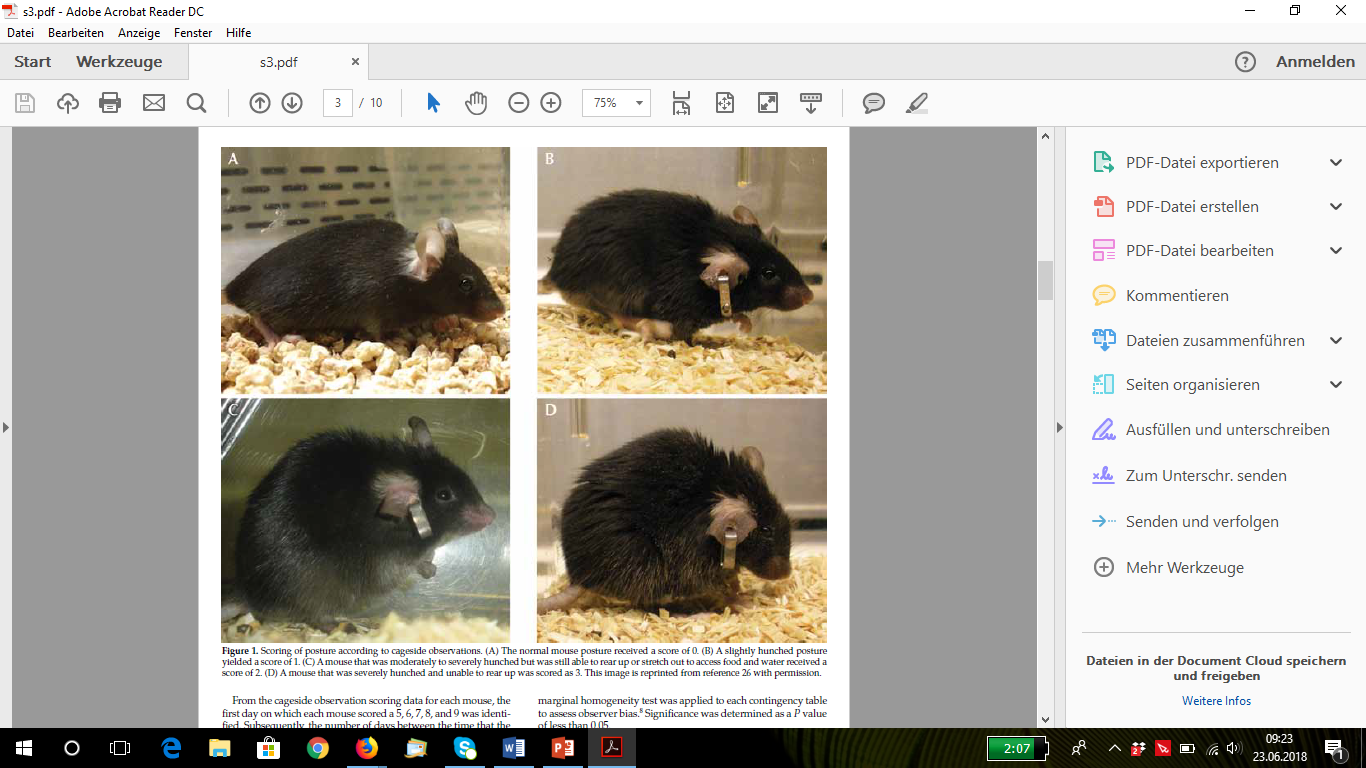


- Non
- Mild
- Moderate
- Severe


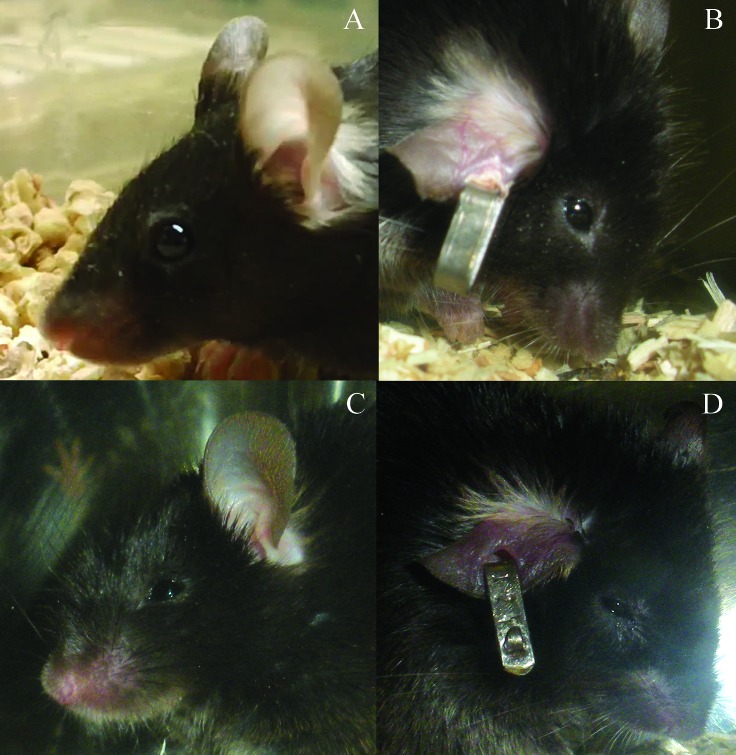


- Non
- Mild
- Moderate
- Severe


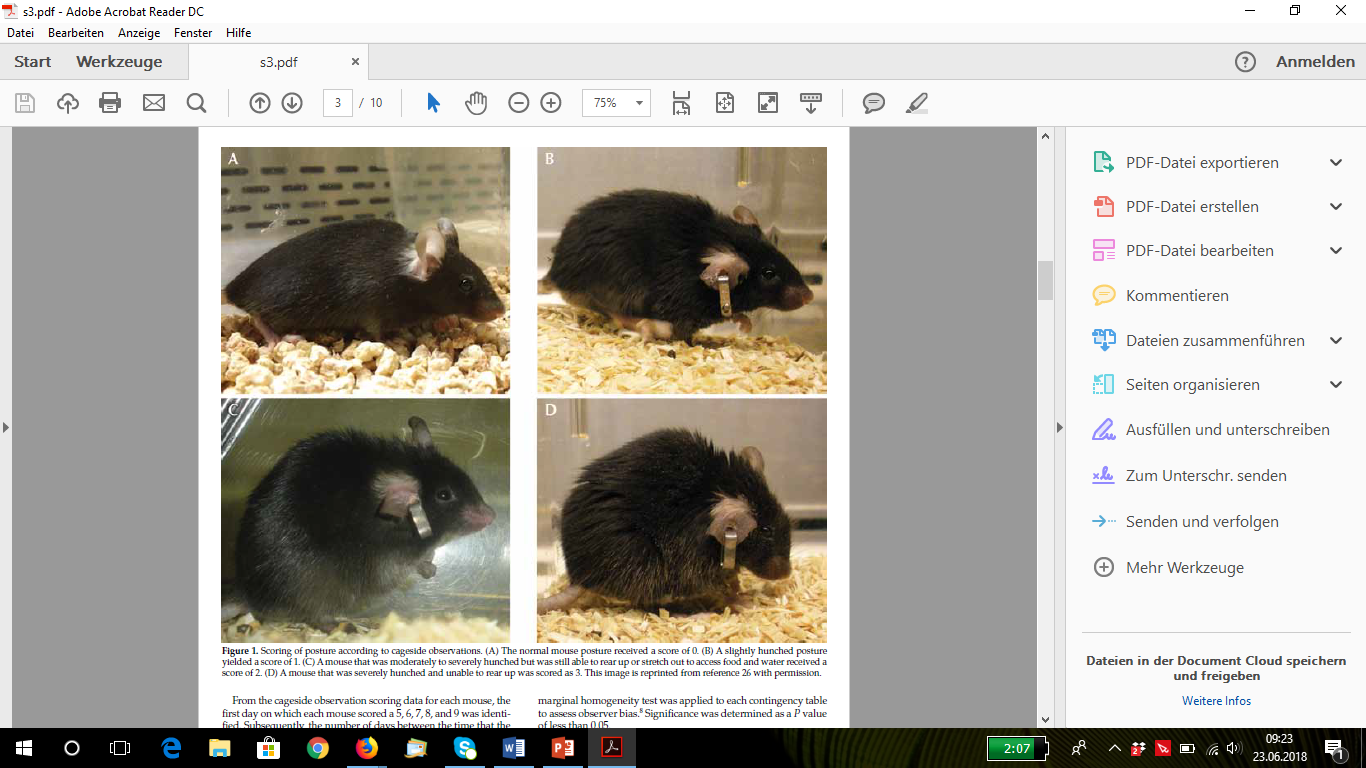


- Non
- Mild
- Moderate
- Severe


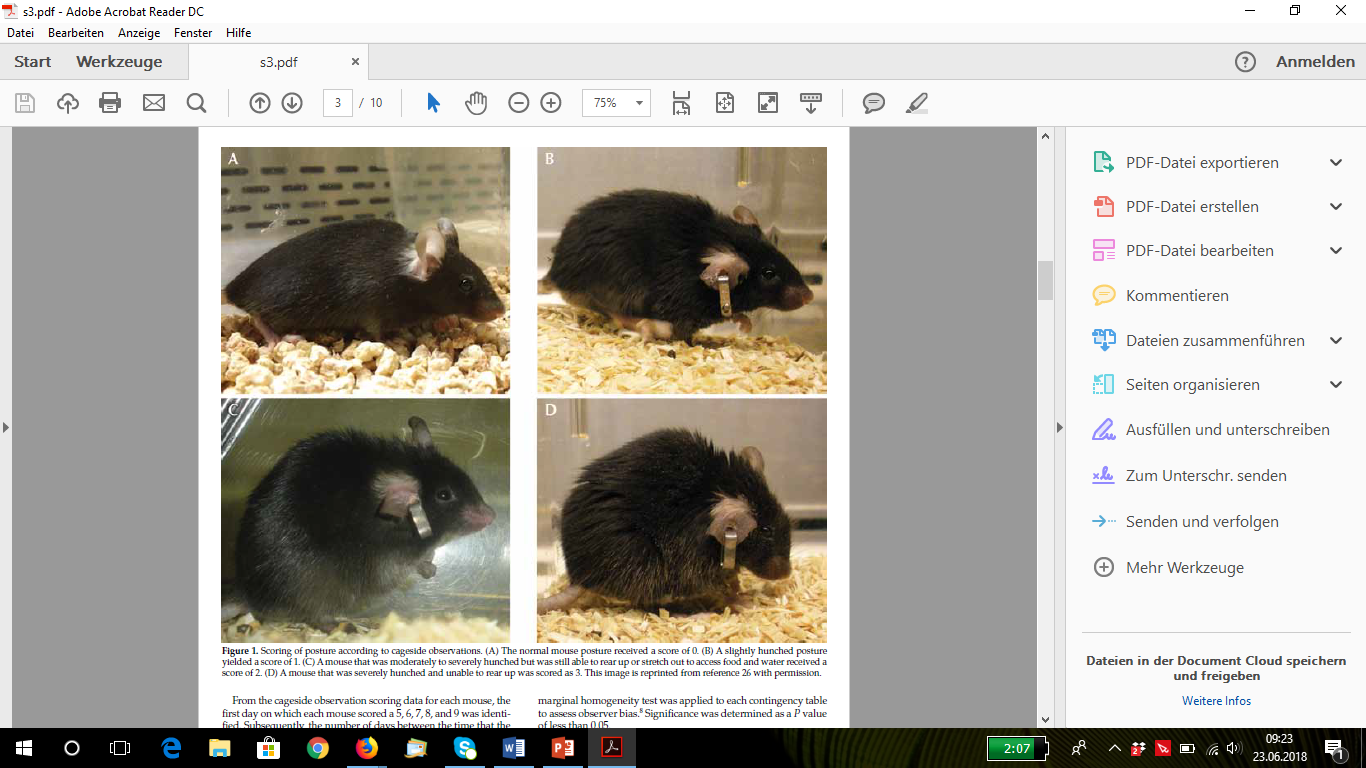


- Non
- Mild
- Moderate
- Severe


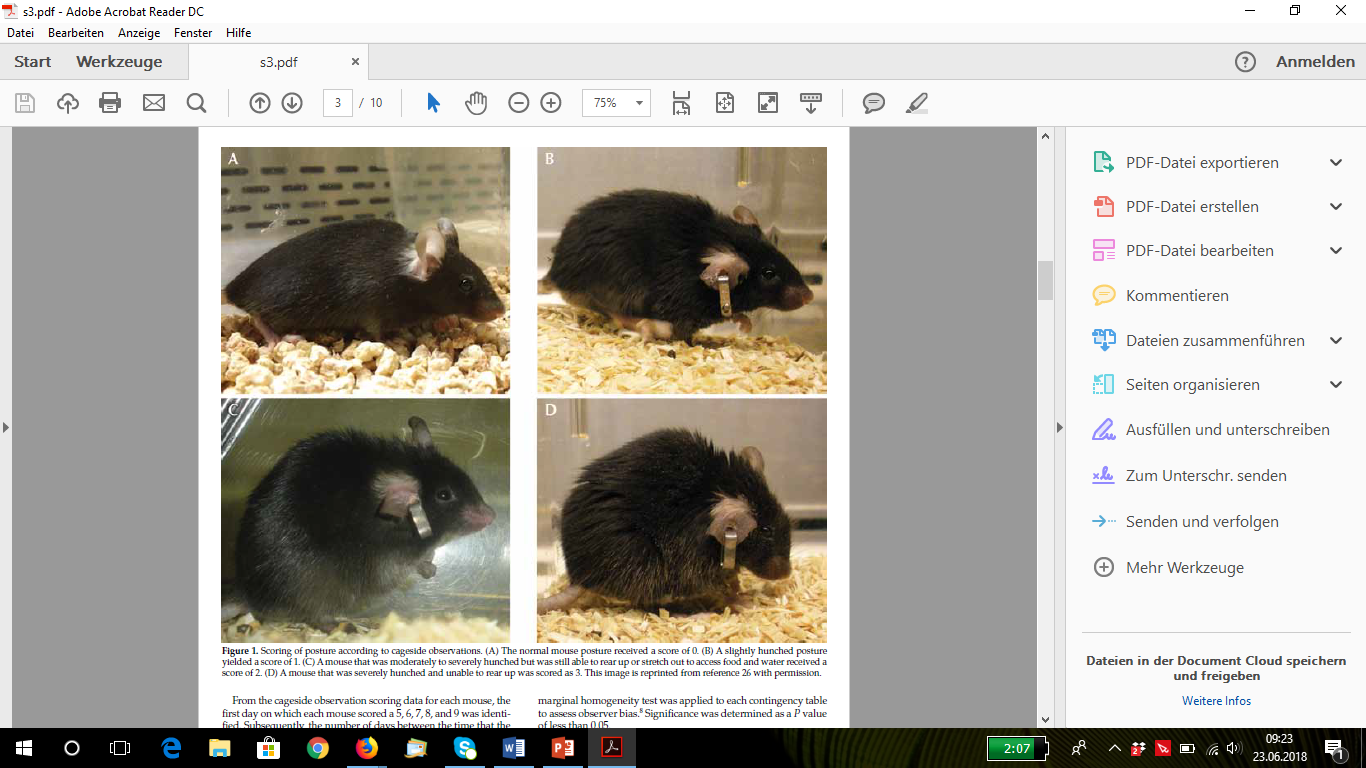


- Non
- Mild
- Moderate
- Severe


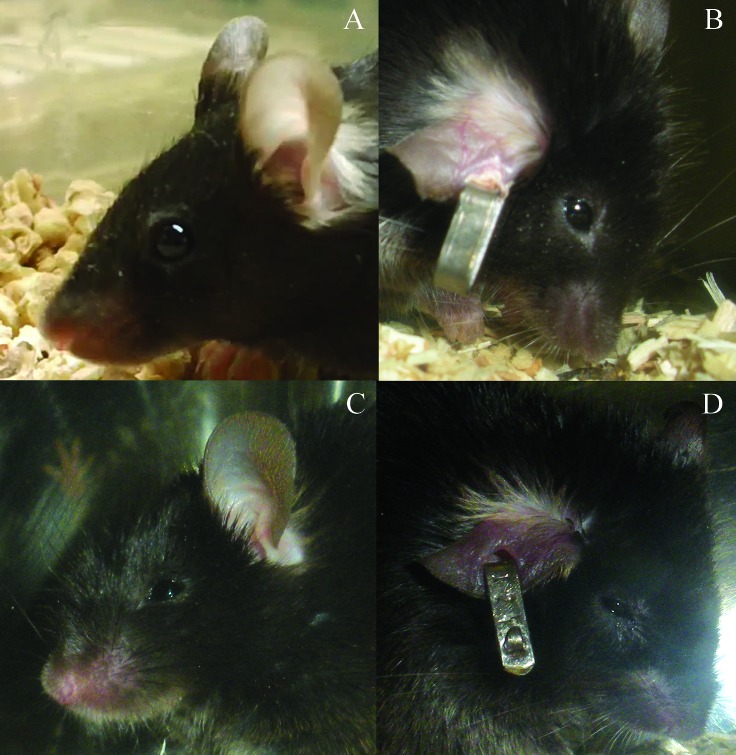


- Non
- Mild
- Moderate
- Severe


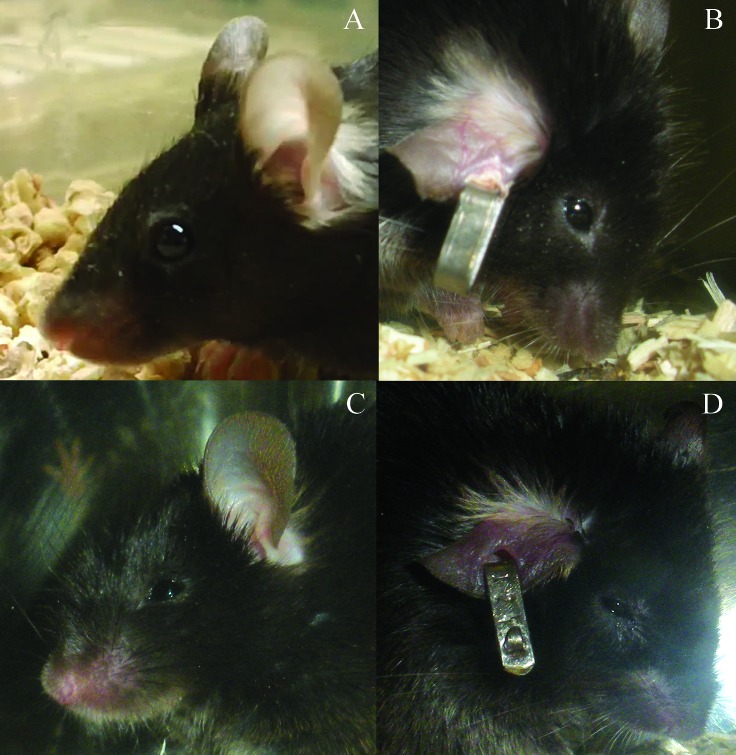


- Non
- Mild
- Moderate
- Severe

Thank you!
