# Supplementary material for: Using photographs for rating severity degrees of clinical appearance in research mice enables valid discrimination of extreme but not mild and moderate conditions: A pilot study
Source: PLoS One. 2023 Nov 2;18(11):e0287965. doi: 10.1371/journal.pone.0287965 (PMC10621849; doi:10.1371/journal.pone.0287965)
Supplement: S1 Table — (DOCX) [file pone.0287965.s002.docx]

| **S2:** **Variables comprising the reference scores.** | | | |
| --- | --- | --- | --- |
| Parameter used | Publication of the score | Score | Score description |
| Eye appearance | [1] | 0 | Normal, open more than 75% |
|  |  | 1 | Open 50%–75% |
|  |  | 2 | Open 25%–49% |
|  |  | 3 | Open less than 25% |
| Orbital tightening | [2] | 0 | Not present |
|  |  | 1 | Moderate |
|  |  | 2 | Severe |
| Cheek bulge |  | 0 | Not present |
|  |  | 1 | Moderate |
|  |  | 2 | Severe |
| Nose bulge |  | 0 | Not present |
|  |  | 1 | Moderate |
|  |  | 2 | Severe |
| Ear position |  | 0 | Not present |
|  |  | 1 | Moderate |
|  |  | 2 | Severe |
| Whisker change |  | 0 | Not present |
|  |  | 1 | Moderate |
|  |  | 2 | Severe |
| Overall MGS |  | 0–12 |  |
| Appearance | [3] | 0 | Normal; coat is smooth, lies flat and often has a sheen; eyes are clear and bright |
|  |  | 1 | Lack of grooming apparent but no other marked changes |
|  |  | 2 | Coat starey; eyes and nose may have discharge |
|  |  | 3 | Coat very starey; external orifices ungroomed, abnormal posture, e.g., may look hunched up, eyes look pale and pupils enlarged |

1. Nunamaker, E.A., et al., *Predictive observation-based endpoint criteria for mice receiving total body irradiation.* Comparative medicine, 2013. **63**(4): p. 313-322.

2. Langford, D.J., et al., *Coding of facial expressions of pain in the laboratory mouse.* Nature methods, 2010. **7**(6): p. 447-449.

3. Morton, D.B. and P.H.M. Griffiths, *Guidelines on the recognition of pain, distress and discomfort in experimental animals and an hypothesis for assessment.* Vet Rec, 1985. **116**(16): p. 431-6.
