# Supplementary material for: Using photographs for rating severity degrees of clinical appearance in research mice enables valid discrimination of extreme but not mild and moderate conditions: A pilot study
Source: PLoS One. 2023 Nov 2;18(11):e0287965. doi: 10.1371/journal.pone.0287965 (PMC10621849; doi:10.1371/journal.pone.0287965)
Supplement: S2 Table — (DOCX) [file pone.0287965.s003.docx]

**S. 3 Table.**

ANOVA for the rating of the different degrees of severity of clinical appearance in images of mice, using the S x P x E x R:E design of the G Theory with interactions

| \| **A: Results for All Images** \| \| \| \| \| \| \| \| \| \| --- \| --- \| --- \| --- \| --- \| --- \| --- \| --- \| --- \| \|  \|  \|  \|  \| Variance Components \| \| \| \| \| \| Source \| SS \| df \| MS \| Random \| Mixed \| Corrected \| % \| SE \| \| E \| 0.057 \| 2 \| 0.028 \| −0.003 \| −0.003 \| −0.002 \| 0.0 \| 0.001 \| \| R:E \| 54.888 \| 81 \| 0.678 \| 0.027 \| 0.085 \| 0.085 \| **5.3** \| 0.016 \| \| S \| 614.01 \| 3 \| 204.7 \| 1.19 \| 1.216 \| 0.912 \| **56.6** \| 0.771 \| \| P \| 14.585 \| 1 \| 14.585 \| 0.030 \| 0.042 \| 0.021 \| 1.3 \| 0.036 \| \| ES \| 3.074 \| 6 \| 0.512 \| 0.006 \| 0.002 \| 0.001 \| 0.1 \| 0.005 \| \| EP \| 0.188 \| 2 \| 0.094 \| −0.001 \| −0.003 \| −0.001 \| 0.0 \| 0.001 \| \| RS:E \| 90.951 \| 243 \| 0.374 \| 0.041 \| 0.187 \| 0.187 \| **11.6** \| 0.021 \| \| RP:E \| 30.603 \| 81 \| 0.378 \| 0.021 \| 0.094 \| 0.094 \| **5.9** \| 0.016 \| \| SP \| 13.35 \| 3 \| 4.45 \| 0.052 \| 0.049 \| 0.019 \| 1.2 \| 0.034 \| \| ESP \| 0.682 \| 6 \| 0.114 \| −0.006 \| −0.006 \| −0.002 \| 0.0 \| 0.002 \| \| RSP:E \| 71.094 \| 243 \| 0.293 \| 0.293 \| 0.293 \| 0.293 \| **18.1** \| 0.026 \| \| Total \| 893.57 \| 671 \|  \|  \|  \|  \| 100% \|  \| |
| --- | --- | --- | --- | --- | --- | --- | --- | --- | --- | --- | --- | --- | --- | --- | --- | --- | --- | --- | --- | --- | --- | --- | --- | --- | --- | --- | --- | --- | --- | --- | --- | --- | --- | --- | --- | --- | --- | --- | --- | --- | --- | --- | --- | --- | --- | --- | --- | --- | --- | --- | --- | --- | --- | --- | --- | --- | --- | --- | --- | --- | --- | --- | --- | --- | --- | --- | --- | --- | --- | --- | --- | --- | --- | --- | --- | --- | --- | --- | --- | --- | --- | --- | --- | --- | --- | --- | --- | --- | --- | --- | --- | --- | --- | --- | --- | --- | --- | --- | --- | --- | --- | --- | --- | --- | --- | --- | --- | --- | --- | --- | --- | --- | --- | --- | --- | --- | --- | --- | --- | --- | --- | --- | --- | --- | --- | --- | --- | --- | --- | --- | --- | --- | --- | --- | --- |
| \| **B: Results for the Images Showing the Extreme Degrees of Severity** \| \| \| \| \| \| \| \| \| \| --- \| --- \| --- \| --- \| --- \| --- \| --- \| --- \| --- \| \|  \|  \|  \|  \| Variance Components \| \| \| \| \| \| Source \| SS \| df \| MS \| Random \| Mixed \| Corrected \| % \| SE \| \| E \| 0.113 \| 2 \| 0.057 \| −0.00200 \| −0.002 \| −0.001 \| 0.0 \| 0.003 \| \| R:E \| 12.875 \| 81 \| 0.159 \| −0.03020 \| 0.01 \| 0.01 \| 0.3 \| 0.012 \| \| S \| 613.440 \| 1 \| 613.440 \| 3.63712 \| 3.65 \| 2.737 \| **91.6** \| 2.981 \| \| P \| 0.107 \| 1 \| 0.107 \| −0.01084 \| −0.006 \| −0.003 \| 0.0 \| 0.01 \| \| ES \| 0.970 \| 2 \| 0.485 \| 0.00398 \| 0.004 \| 0.002 \| 0.1 \| 0.006 \| \| EP \| 0.018 \| 2 \| 0.009 \| −0.00217 \| −0.002 \| −0.001 \| 0.0 \| 0.001 \| \| RS:E \| 19.589 \| 81 \| 0.242 \| 0.08510 \| 0.121 \| 0.121 \| 4.0 \| 0.02 \| \| RP:E \| 8.875 \| 81 \| 0.11 \| 0.01896 \| 0.037 \| 0.037 \| 1.2 \| 0.010 \| \| SP \| 2.012 \| 1 \| 2.012 \| 0.02285 \| 0.023 \| 0.009 \| 0.3 \| 0.02 \| \| ESP \| 0.185 \| 2 \| 0.092 \| 0.00074 \| 0.001 \| 0.000 \| 0.0 \| 0.002 \| \| RSP:E \| 5.804 \| 81 \| 0.072 \| 0.07165 \| 0.072 \| 0.072 \| 2.4 \| 0.011 \| \| Total \| 663.988 \| 335 \|  \|  \|  \|  \| 100% \|  \| |
| \| **C. Results for the** **Images Showing the Intermediate Degrees of Severity** \| \| \| \| \| \| \| \| \| \| --- \| --- \| --- \| --- \| --- \| --- \| --- \| --- \| --- \| \|  \|  \|  \|  \| Variance Components \| \| \| \| \| \| Source \| SS \| df \| MS \| Random \| Mixed \| Corrected \| % \| SE \| \| E \| 0.113 \| 2 \| 0.057 \| −0.012 \| −0.009 \| −0.006 \| 0.0 \| 0.007 \| \| R:E \| 70.563 \| 81 \| 0.871 \| 0.055 \| 0.152 \| 0.152 \| **13.2** \| 0.049 \| \| S \| 0.003 \| 1 \| 0.003 \| −0.006 \| −0.003 \| −0.002 \| 0.0 \| 0.004 \| \| P \| 25.741 \| 1 \| 25.741 \| 0.151 \| 0.151 \| 0.075 \| **6.6** \| 0.125 \| \| ES \| 1.935 \| 2 \| 0.967 \| 0.015 \| 0.008 \| 0.004 \| 0.3 \| 0.012 \| \| EP \| 0.554 \| 2 \| 0.277 \| 0.002 \| −0.002 \| −0.001 \| 0.0 \| 0.004 \| \| RS:E \| 42.813 \| 81 \| 0.529 \| 0.026 \| 0.264 \| 0.264 \| **23.0** \| 0.055 \| \| RP:E \| 48.455 \| 81 \| 0.598 \| 0.061 \| 0.180 \| 0.180 \| **15.6** \| 0.059 \| \| SP \| 0.074 \| 1 \| 0.074 \| 0.000 \| −0.005 \| −0.002 \| 0.0 \| 0.001 \| \| ESP \| 0.113 \| 2 \| 0.057 \| −0.015 \| −0.015 \| −0.004 \| 0.0 \| 0.003 \| \| RSP:E \| 38.563 \| 81 \| 0.476 \| 0.476 \| 0.476 \| 0.476 \| 41.3 \| 0.074 \| \| Total \| 228.926 \| 335 \|  \|  \|  \|  \| 100% \|  \| |

Abbreviations for S3:

SS, sum of squares; df, degrees of freedom; MS, mean squares; SE, standard errors; E, experience of rater; R:E, rater (nested in experience); S, severity degree of pictured mouse; P, perspective; ES, interaction of experience of rater and severity degree of pictured mouse; EP, interaction of experience of rater and perspective; RS:E, interaction of rater and severity degree of pictured mouse; RP:E, interaction of rater and perspective; SP, interaction of severity degree of pictured mouse and perspective; ESP, interaction of experience, severity degree of pictured mouse and perspective; RSP:E, interaction of severity degree of pictured mouse and perspective. In bold: values of special interest.
